# Association Between Patient Behavior and Clinician Emotional Burden, Perceived Performance, and Decision‐Making in Implant Dentistry: A Cross‐Sectional Study Among Clinicians

**DOI:** 10.1002/cre2.70450

**Published:** 2026-09-08

**Authors:** Aspasia Pachiou, Viviane Laura Humm, Nadja Naenni, Eleana Kontonasaki, Ronald E. Jung, Daniel S. Thoma

**Affiliations:** ^1^ Clinic of Reconstructive Dentistry, Center for Dental Medicine University of Zurich Zurich Switzerland; ^2^ Department of Prosthodontics, School of Dentistry Aristotle University of Thessaloniki Thessaloniki Greece; ^3^ Department of Periodontology, Research, Institute for Periodontal Regeneration Yonsei University College of Dentistry Seoul Republic of Korea

**Keywords:** decision‐making, dentist–patient communication, emotional burden, patient behavior

## Abstract

**Objectives:**

To evaluate the association between patient behavior and clinician emotional burden, perceived clinical performance, interaction difficulty, and decision‐making in implant dentistry.

**Material and Methods:**

A cross‐sectional online questionnaire was distributed via individual invitations to 150 dental professionals involved in implant dentistry. A total of 127 clinicians completed the survey (response rate: 84.7%). The questionnaire assessed demographic and professional characteristics, clinicians’ perceptions of patient behavior, emotional responses, and the perceived impact of patient‐related factors on clinical performance and decision‐making. Composite domains for emotional burden and interaction difficulty were developed, and their internal consistency was assessed using Cronbach's *α*. Associations were evaluated using Spearman's correlation and linear regression analyses.

**Results:**

More challenging patient behavior was significantly associated with increased emotional burden (*ρ* = 0.50, *p* < 0.001) and interaction difficulty (*ρ* = 0.46, *p* < 0.001). Similar findings were observed when patient behavior was compared with usual clinical cases (both *p* < 0.001). Procedural difficulty showed only weak or non‐significant associations with emotional burden and interaction difficulty. Emotional burden was strongly associated with interaction difficulty (*ρ* = 0.84, *p* < 0.001), avoidance of scheduling similar patients (*ρ* = 0.47, *p* < 0.001), and lower perceived clinical performance (*ρ* = 0.27, *p* = 0.002). Regression analyses confirmed that patient behavior was significantly associated with emotional burden (*p* < 0.001).

**Conclusions:**

Patient behavior appears to be associated with clinician emotional burden and perceived clinical performance more strongly than case difficulty in implant dentistry.

## 1 Introduction

Implant dentistry is widely recognized as a predictable and evidence‐based treatment modality for the rehabilitation of partially and fully edentulous patients, demonstrating high survival and success rates across a broad range of clinical indications (Jung et al. 2012; Kupka et al. 2024). Advances in digital technologies, biomaterials, and surgical protocols have further enhanced treatment precision and long‐term outcomes. Nevertheless, the success of implant therapy extends beyond technical execution and biological integration, encompassing behavioral, psychological, and human‐factor dimensions that influence clinical performance and decision‐making (Staedt et al. 2020; Papaspyridakos et al. 2012).

The impact of stress and mental workload on clinical performance has been well documented in health care (Tam et al. 2025; Arora et al. 2010). Elevated cognitive load has been associated with impaired decision‐making, reduced efficiency, and an increased likelihood of errors (Arora et al. 2010). In dentistry, clinician‐related stressors such as time pressure, case difficulty, and ergonomic strain have been shown to negatively affect both performance and well‐being (Rada and Johnson‐Leong 2004; Kulkarni et al. 2016). Furthermore, emotional responses such as frustration, anxiety, and interpersonal tension may interfere with attentional control and clinical focus, particularly during technically demanding procedures (Zuercher et al. 2026).

The clinician's performance is determined by a combination of task demands, environmental conditions, and interpersonal dynamics (Audet et al. 2025; Wenghofer et al. 2009). The patient's behavior represents a context‐specific factor that may shape clinicians’ responses during treatment (Wenghofer et al. 2009). In implant dentistry, where surgical and prosthetic procedures require sustained concentration and precision, such variability may introduce challenges beyond technical complexity and prompt adaptations in treatment approach. Notably, evidence from practice‐based research highlights the importance of predictability and a trusting patient–clinician relationship in everyday prosthodontic practice (Pachiou et al. 2026). Furthermore, patients’ preferences for involvement in prosthodontic treatment decision‐making have been shown to vary and evolve throughout treatment, emphasizing the importance of effective patient–clinician communication (Strauss et al. 2026). Similarly, a recent cross‐sectional study among Norwegian dentists and dental students reported that greater exposure to challenging patient encounters was associated with lower quality of life, highlighting the broader impact of these interactions on dental professionals (Johnsen et al. 2023). Together, these findings further emphasize the clinical relevance of interpersonal dynamics and the need to better understand their impact on clinician performance and decision‐making.

Despite this relevance, there is a lack of structured evidence examining how such patient‐related factors influence clinician performance, emotional burden, and decision‐making in implant dentistry. To address this gap, a structured questionnaire was developed to capture clinician‐reported perceptions of case difficulty, emotional responses, and the perceived impact of patient‐related factors on clinical outcomes and decision‐making in implant clinical practice.

In the present study, the term “challenging patient encounter” refers to the clinician's perception of an interaction in which patient‐related behaviors complicated communication or treatment delivery. This perception does not imply that the patient alone was responsible for the difficulty of the encounter, but rather reflects the clinician's experience of the interaction.

Therefore, the aim of this cross‐sectional study was to evaluate the association between clinicians’ perceptions of patient behavior and their emotional burden, perceived clinical performance, and decision‐making in implant dentistry using a structured questionnaire administered to clinicians involved in implant dentistry. It was hypothesized that clinicians’ perceptions of more challenging patient behavior would be associated with increased emotional burden during implant‐related procedures.

## 2 Materials and Methods

This cross‐sectional questionnaire‐based study was conducted between December 2025 and March 2026 to investigate the perceived impact of patient behavior on clinicians’ performance, emotional burden, and decision‐making in implant dentistry. The study was performed in accordance with the principles of the Declaration of Helsinki. Participation was voluntary, and the survey was administered in a fully anonymous format. No personally identifiable data were collected. According to Swiss legislation (Human Research Act), research involving fully anonymized questionnaire data without identifiable personal information does not require approval from a cantonal ethics committee. Therefore, formal ethical approval was not required for this study.

The questionnaire was administered electronically using the LimeSurvey platform. Individual invitations containing the survey link were sent to 150 dental professionals involved in implant dentistry through the authors’ professional networks. The recruitment target was determined pragmatically based on the number of substantive questionnaire items contributing to the clinician‐reported constructs, excluding demographic and descriptive questions, using an approximate target of five invited participants per substantive item, while also considering the size of the accessible professional network. Of the invited clinicians, 127 clinicians completed the survey (response rate: 84.7%).

Eligible participants included dental professionals actively involved in implant dentistry, including general dentists, residents, and specialists in prosthodontics, implantology, periodontology, and oral surgery. Participants were recruited using a convenience sampling approach through the authors’ professional networks. Inclusion criteria required clinical experience in implant treatment planning and/or surgical or prosthetic implant procedures. Responses from individuals without implant‐related clinical experience, as well as incomplete questionnaires, were excluded from the final analysis.

The questionnaire was specifically developed for the present study following a literature‐informed review of factors related to patient behavior, clinician stress, emotional burden, and clinical decision‐making in health care and dental practice. The instrument captured multiple dimensions of the clinician experience, including demographic and professional characteristics, perceived case difficulty, patient behavior, and clinician‐reported outcomes. Participants were asked to recall and evaluate a specific implant patient encounter they had experienced and to rate the extent to which patient‐related factors influenced clinical outcomes (subjective and objective), decision‐making, treatment time, efficiency, clinical focus, and subsequent patient management. Participants were instructed to reflect on their most recent implant patient they perceived as challenging. The term “challenging” was intentionally broad and could encompass technical, communication‐related, interpersonal, or other aspects of the clinical encounter. Patient behavior was subsequently assessed as a separate questionnaire item based on the clinician's perception of the encounter, allowing respondents to independently rate the patient's cooperativeness regardless of the reason the encounter was perceived as challenging. In addition, items assessed emotional and interpersonal responses, including clinicians’ perceived communication difficulty, tension, frustration, dissatisfaction, and interpersonal challenges during the interaction. All items were recorded using 5‐point Likert‐type scales. The complete questionnaire is provided in the Supplementary Material. The questionnaire was developed and administered in English, as English is widely used in postgraduate education, scientific communication, and continuing professional education in implant dentistry.

Prior to distribution, the questionnaire underwent face validity assessment by a panel of five experienced clinicians in implant dentistry, who evaluated the clarity, relevance, comprehensiveness, and clinical applicability of the items. Minor revisions were subsequently made to improve wording and structure. Questionnaire items were developed following a review of the relevant literature and were organized into predefined domains of interest, including patient behavior, perceived clinical impact, emotional burden, and treatment‐related decision‐making. Internal consistency of the composite domains was assessed using Cronbach's *α*. Test–retest reliability assessment was not feasible due to the fully anonymous study design. Although the questionnaire underwent preliminary expert review, formal psychometric validation, including comprehensive assessment of content validity, was beyond the scope of this exploratory study. Therefore, the questionnaire should not be considered a formally validated psychometric instrument.

### 2.1 Statistical Analysis

For analytical purposes, conceptually related items were grouped into predefined composite domains. Two primary domains were defined: (1) emotional burden associated with the patient encounter, reflecting negative emotional responses during treatment, and (2) interaction difficulty, reflecting perceived interpersonal challenges during the clinical encounter. Composite scores were calculated by averaging item responses within each domain, with higher scores indicating greater emotional burden or perceived interaction difficulty.

The internal consistency of multi‐item domains was assessed using Cronbach's *α* coefficient. Values ≥ 0.70 were considered indicative of acceptable reliability. Domains demonstrating insufficient internal consistency were not retained as composite variables and were instead analyzed at the individual item level.

Data were collected and managed electronically using LimeSurvey and analyzed using RStudio (R Foundation for Statistical Computing, Vienna, Austria). Descriptive statistics were calculated for all variables. Categorical variables were summarized as frequencies and percentages, while ordinal and composite variables were presented as means and standard deviations.

Associations between variables were assessed using Spearman's rank correlation coefficients, given the ordinal nature of the data. Linear regression analyses were performed to evaluate the association between patient behavior and emotional burden, as well as to assess the independent effects of patient behavior and case difficulty on emotional burden. Additional regression analyses were conducted to examine the association between emotional burden and avoidance behavior.

Comparisons between groups (such as gender) were performed using the Wilcoxon rank‐sum test due to the non‐parametric distribution and ordinal scaling of the data. Gender‐based comparisons were conducted among participants who reported their gender.

All statistical tests were two‐sided, and a significance level of *p* < 0.05 was considered statistically significant.

## 3 Results

### 3.1 Descriptive Statistics

A total of 127 dentists completed the questionnaire, corresponding to a response rate of 84.7% (127/150). The demographic and professional characteristics of the study population are summarized in Table 1.

**Table 1:** Demographic and Professional Characteristics of the Study Population (*n* = 127).

Variable	Category	*n* (%)
Clinical role	General dentist	60 (47.2)
	Resident	15 (11.8)
	Specialist (perio/oral surgery)	24 (18.9)
	Specialist (prostho/implant)	22 (17.3)
	Other	6 (4.7)
Years in dentistry	< 3 years	0 (0.0)
	3–5 years	35 (27.8)
	6–10 years	55 (43.7)
	11–20 years	22 (17.5)
	> 20 years*	14 (11.1)
Work setting	Private practice	86 (67.7)
	Academic	33 (26.0)
	Hospital	6 (4.7)
	Other	2 (1.6)
Monthly implant cases	1–2	50 (39.4)
	3–5	31 (24.4)
	5–10	17 (13.4)
	> 10	29 (22.8)
Age	< 30	25 (19.7)
	30–39	78 (61.4)
	40–49	14 (11.0)
	50–59	9 (7.1)
	≥ 60	1 (0.8)
Gender	Female	65 (51.2)
	Male	61 (48.0)
	Prefer not to say	1 (0.8)

* One participant did not respond to this question; percentages are based on the 126 available responses. Percentages may not total 100% because of rounding.

The sample consisted predominantly of clinicians in early to mid‐career stages, with most participants reporting 6–10 years of clinical experience. The majority worked in private practice and reported a low to moderate monthly implant caseload, with approximately 60% performing 1–5 implant procedures per month. The study population was balanced in terms of gender distribution and primarily comprised clinicians aged 30–39 years.

The survey population was multi‐country, with the largest proportion of respondents practicing in Switzerland (41.73%), followed by Greece (19.69%) and the United Kingdom (9.45%).

Regarding the index clinical encounter, nearly half of clinicians rated case difficulty as neutral (48.82%), while approximately one‐third (32.28%) considered the case difficult or very difficult. Patient behavior was perceived as cooperative by 45.67% of respondents, whereas 40.16% reported somewhat difficult or difficult behavior.

Most clinicians reported satisfaction with their clinical performance (70.87% satisfied or very satisfied). Patient‐related factors were perceived to influence multiple aspects of care, including treatment time (74.02%), perceived efficiency (72.44%), clinical decision‐making (59.84%), and subjective treatment outcomes (63.78%). Emotional exhaustion was reported to be at least moderately affected in 78.75% of cases.

Substantial emotional and interpersonal burden was evident. Nearly half of clinicians reported feeling tense during the interaction (47.24%), while 34.65% reported difficulty communicating with the patient. Notably, 62.21% reported feeling glad when the appointment ended, and 53.54% perceived the patient as difficult to work with.

**Figure 1:**
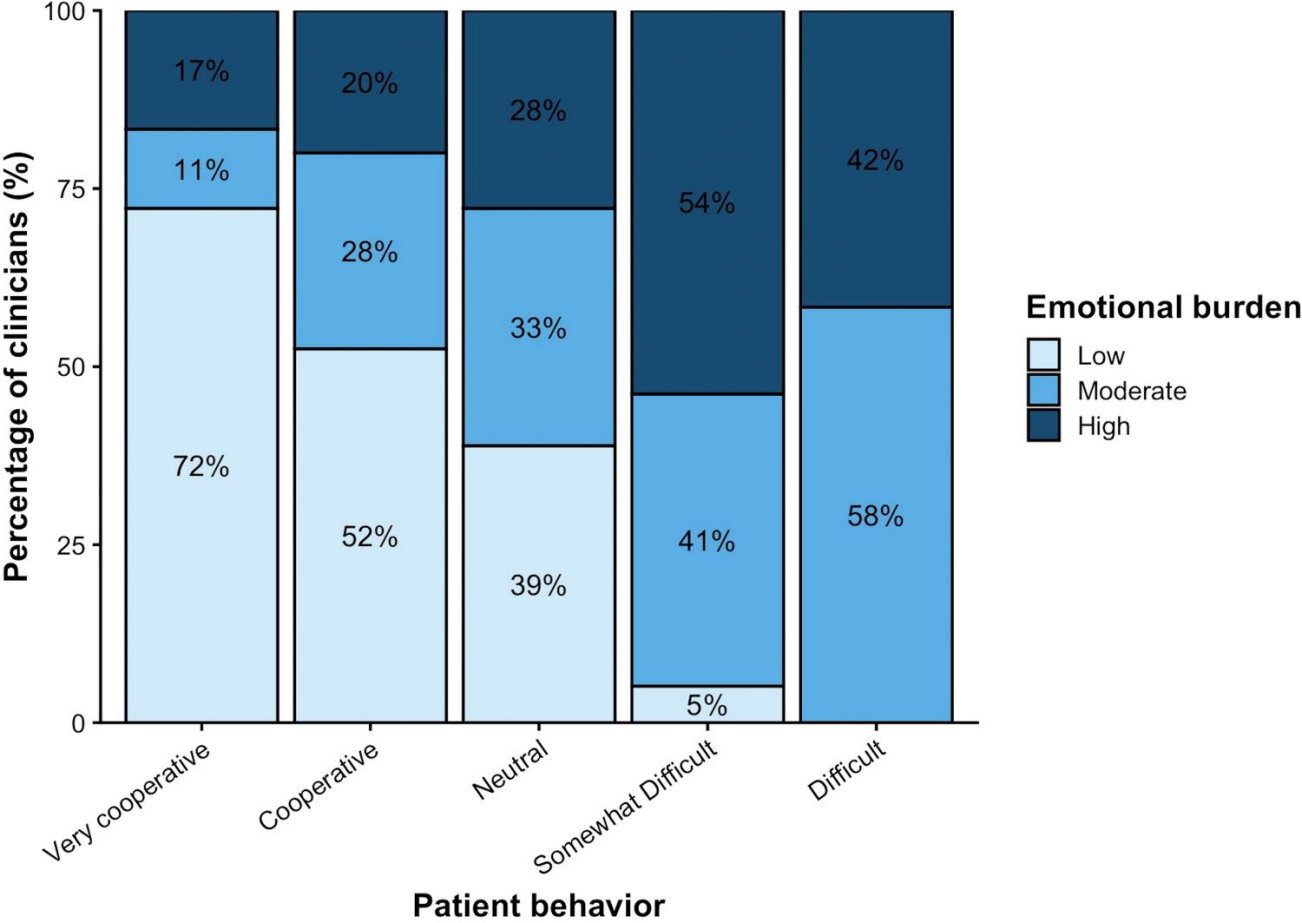
Distribution of emotional burden levels according to patient behavior.

As illustrated in Figure 1, emotional burden increased progressively with worsening patient behavior. While the majority of clinicians reported low emotional burden in very cooperative encounters (72%), high emotional burden became predominant in somewhat difficult (54%) and difficult (42%) interactions.

**Figure 2:**
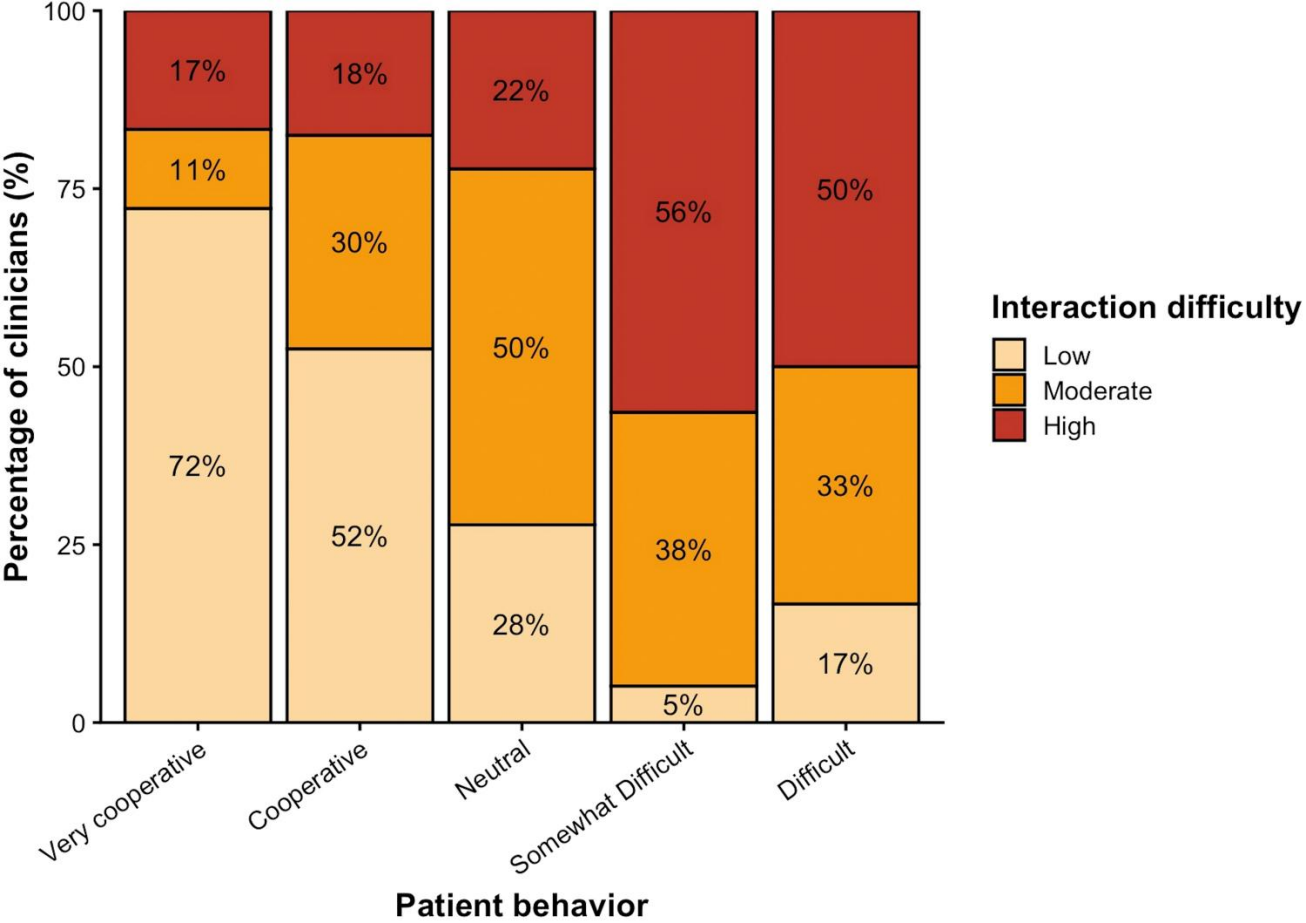
Distribution of interaction difficulty according to patient behavior.

A similar trend was observed for interaction difficulty (Figure 2), with low difficulty reported in 72% of very cooperative encounters, shifting to predominantly high difficulty in somewhat difficult (56%) and difficult (50%) patient interactions.

**Figure 3:**
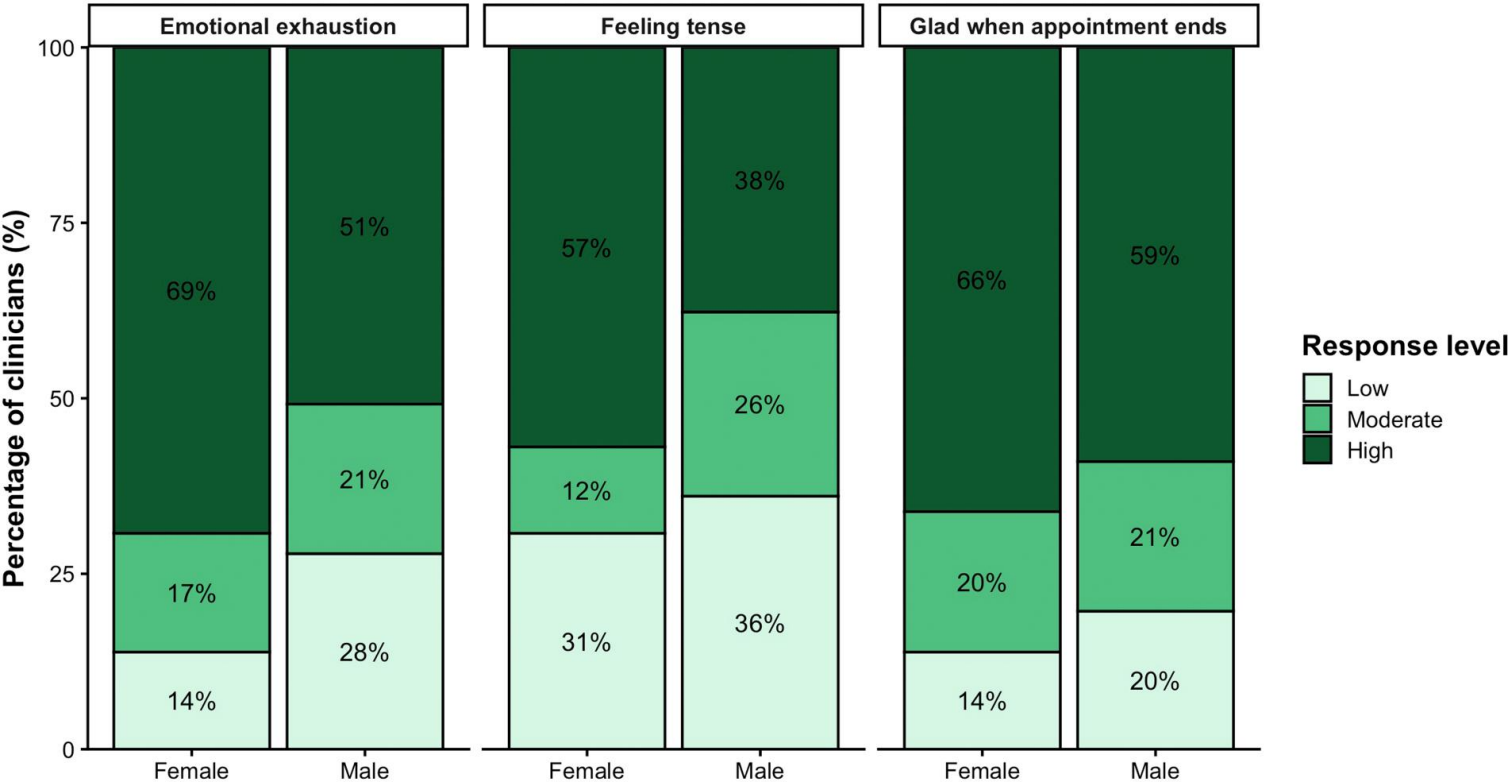
Gender differences in selected emotional responses.

Gender‐based differences in specific emotional responses are presented in Figure 3. Female clinicians reported slightly higher levels of emotional exhaustion and tension, whereas male clinicians more frequently reported being glad at the end of the appointment, although overall patterns were comparable between groups.

A detailed distribution of responses across all variables is presented in Table 2.

**Table 2:** Distribution of responses across clinical and emotional variables (%).

Variable	Low (%)	Moderate (%)	High (%)
Emotional exhaustion	21.26	18.90	59.85
Feeling tense	33.86	18.90	47.24
Clinical focus affected	42.52	27.56	29.92
Treatment time affected	25.99	31.50	42.52
Efficiency affected	27.55	36.22	36.22
Decision‐making affected	40.16	31.50	28.34
Approach to next patient	50.40	30.71	18.90
Non‐compliance decisions	33.86	29.92	36.22
Avoidance tendency	46.45	25.20	28.34
Glad when appointment ends	16.53	21.26	62.21
Difficult to work with	27.56	18.90	53.54

Internal consistency analysis demonstrated good reliability for the emotional burden scale (Cronbach's *α* = 0.88) and excellent reliability for the interaction difficulty scale (Cronbach's *α* = 0.92).

### 3.2 Correlation Analysis

Spearman correlation analysis suggested that perceived patient behavior was significantly associated with both emotional burden (*ρ* = 0.50, *p* < 0.001) and interaction difficulty (*ρ* = 0.46, *p* < 0.001). Similar results were observed for patient behavior compared to usual cases (*ρ *= 0.51 and *ρ* = 0.43, respectively; both *p* < 0.001).

In contrast, case difficulty showed only weak associations with emotional burden (*ρ* = 0.17, *p* = 0.049) and was not significantly associated with interaction difficulty (*p* = 0.16).

Emotional burden and interaction difficulty were strongly correlated (*ρ* = 0.84, *p* < 0.001), indicating substantial overlap between these constructs. Higher emotional burden was also associated with increased avoidance of scheduling similar patients (*ρ* = 0.47, *p* < 0.001) and lower perceived clinical performance (*ρ* = 0.27, *p* = 0.002).

### 3.3 Regression Analysis

A multiple linear regression analysis was performed to evaluate the independent effects of patient behavior and case difficulty on emotional burden. Patient behavior was significantly associated with emotional burden (*β* = 0.38, *p* < 0.001), whereas case difficulty was not (*β* = 0.03, *p* = 0.759). The overall model was statistically significant (*F*(2,124) = 21.82, *p* < 0.001) and explained 26% of the variance in emotional burden (*R* ^2^ = 0.26).

A secondary model using patient behavior relative to usual cases suggested an even stronger association (*β* = 0.52, *p* < 0.001), with case difficulty again showing no significant effect (*β* = 0.07, *p* = 0.441). This model explained 27% of the variance in emotional burden (*R* ^2^ = 0.27).

Additionally, linear regression analysis showed that emotional burden was significantly associated with avoidance of scheduling similar patients (*β* = 0.59, *p* < 0.001), explaining 21% of the variance in avoidance behavior (*R* ^2^ = 0.21).

### 3.4 Gender Differences

Exploratory analyses revealed no significant differences between genders in emotional burden (*p* = 0.19) or perceived interaction difficulty (*p* = 0.45). However, a statistically significant difference was observed in avoidance behavior (*p* = 0.012), with female clinicians reporting higher levels of avoidance (mean = 2.92, SD = 1.15) compared to male clinicians (mean = 2.38, SD = 1.19).

## 4 Discussion

The present study highlights the potential relevance of patient behavior in implant dentistry by demonstrating that encounters perceived by clinicians as challenging were associated with increased emotional burden and perceived interaction difficulty. Notably, patient behavior showed stronger associations with emotional burden than case difficulty, emphasizing the importance of interpersonal and human‐factor dimensions in implant practice. Although patient behavior was not directly associated with lower perceived clinical performance, greater emotional burden was associated with lower perceived clinical performance, suggesting that interpersonal factors may influence clinicians’ experiences indirectly. While technical complexity is traditionally regarded as the primary challenge in implant dentistry, the present findings suggest that interpersonal patient‐related factors may represent an additional and underrecognized source of clinician burden. Overall, the findings support the study hypothesis that more challenging patient behavior is associated with greater emotional burden among clinicians involved in implant dentistry. To the authors’ knowledge, studies specifically examining the influence of patient behavior on clinicians’ emotional burden and decision‐making in implant dentistry are lacking. Therefore, comparisons with the existing literature necessarily rely on evidence from general dentistry and other healthcare settings.

A central finding of this study is the robust association between challenging patient behavior and increased emotional burden, including tension, frustration, and emotional exhaustion. Increased emotional burden was also associated with lower perceived clinical performance. These findings are consistent with studies in general dentistry and primary care showing that difficult patient encounters constitute an important source of occupational stress and emotional burden for clinicians (Lin et al. 1991; Myers and Myers 2004; Alvenfors et al. 2022; An et al. 2009). Similarly, qualitative work among dental professionals has highlighted communication difficulties, unrealistic expectations, and emotionally demanding interactions as key contributors to challenging patient encounters (Alvenfors et al. 2022). In dentistry specifically, psychosocial stressors, such as time pressure, patient anxiety, and complex communication, have been identified as key contributors to professional strain and burnout (Ayers et al. 2009; Stanley et al. 2024). Given that implant dentistry often involves technically demanding procedures requiring sustained concentration and precision, the impact of such emotional strain may be particularly pronounced in this field.

The strong correlation observed between emotional burden and interaction difficulty suggests substantial overlap between these constructs. This finding is not unexpected, as emotionally demanding patient encounters are likely to be perceived as more challenging interpersonal interactions. Nevertheless, the two composite measures were intended to capture complementary aspects of the clinician experience: emotional burden reflects clinicians’ affective responses to the encounter, whereas interaction difficulty reflects perceived interpersonal challenges during the clinical encounter. Future studies using formally validated psychometric instruments may further clarify the distinction between these related constructs.

Interestingly, despite the presence of challenging interactions, patient behavior was not directly associated with perceived clinical performance. Clinicians consistently reported high levels of satisfaction with their performance, suggesting a notable degree of professional resilience (An et al. 2013). This finding aligns with previous research indicating that experienced clinicians are often able to maintain technical standards even under stressful conditions, likely due to training, procedural familiarity, and cognitive coping strategies (An et al. 2013; LeBlanc 2009). However, this apparent resilience should be interpreted cautiously, as sustained exposure to stress has been shown to negatively affect cognitive function, decision‐making, and long‐term performance (Arora et al. 2010; Chipchase et al. 2017).

One of the most important contributions of the present study is the finding that patient behavior was associated with perceived clinical impact in a pattern consistent with an indirect possible relationship through emotional burden. This conceptual possible relationship suggests that patient‐related stressors do not directly compromise technical execution but may influence the clinicians’ perceptions thereof, the decision‐making processes, and the overall clinical experience (Chipchase et al. 2017; Croskerry et al. 2013). Such findings are consistent with cognitive load theory, which posits that emotional stress can consume attentional resources and indirectly affect performance‐related processes (LeBlanc 2009). In the context of implant dentistry, this may translate into altered treatment planning, reduced efficiency, or increased perceived difficulty, even when objective outcomes remain unaffected.

The descriptive findings further reinforce this interpretation. A substantial proportion of clinicians reported that patient‐related factors influenced treatment time, efficiency, and decision‐making, while many also reported feeling glad at the end of appointments. These observations highlight the practical relevance of patient behavior in everyday clinical settings and suggest that its impact extends beyond measurable clinical outcomes to include workflow, clinician experience, and perceived workload (Weigl et al. 2017). Such findings are in line with previous reports indicating that patient cooperation and communication significantly affect procedural efficiency and clinician stress levels in dental practice (Armfield and Heaton 2013; Ho et al. 2025).

Gender‐based analyses did not reveal statistically significant differences in emotional responses, suggesting that the perceived impact of patient behavior on emotional burden is broadly consistent across male and female clinicians. This finding contrasts with some studies in health care reporting higher burnout rates among female practitioners (Purvanova and Muros 2010; Man et al. 2026) but aligns with others indicating that workplace stressors may affect clinicians similarly when professional roles and environments are comparable (Fida et al. 2023). The lack of significant gender differences in the present study suggests that the observed associations were broadly consistent across male and female clinicians, although confirmation in more diverse and representative populations is warranted.

From a clinical perspective, these findings have important implications. Patient behavior was significantly associated with emotional burden, whereas case difficulty showed weak or non‐significant associations in multivariable models. Although clinicians reported maintaining their clinical performance despite challenging patient behavior, the associated emotional burden may accumulate over time and contribute to chronic stress and burnout (Moro et al. 2022). Similarly, limited evidence from implant dentistry has identified burnout as a relevant concern among implant clinicians (Salgado‐Peralvo et al. 2025). Burnout has been widely documented among dental professionals and is associated with decreased job satisfaction, reduced quality of care, and increased risk of errors (Kulkarni et al. 2016; Anishchuk and Seery 2025). Therefore, addressing the psychological and interpersonal dimensions of implant dentistry is essential. Interventions such as communication training, behavioral management strategies, and stress reduction programs may help mitigate the perceived impact of difficult patient interactions and improve clinician well‐being (Croskerry et al. 2013; Negucioiu et al. 2024; Boissy et al. 2016).

These findings also support integrating non‐technical skills into implant education. Training programs traditionally emphasize surgical and prosthetic competence, whereas structured education in communication, conflict management, and emotionally challenging patient encounters remains limited. Addressing these competencies may help clinicians navigate difficult interactions more effectively while maintaining both clinician well‐being and high‐quality patient care.

The present study has several notable strengths. First, it addresses an underexplored aspect of implant dentistry by focusing on the interaction between patient behavior and clinician experience, thereby expanding the current understanding of non‐technical factors in clinical performance. To capture these implant‐specific constructs, a study‐specific questionnaire was developed. Although validated instruments are available to assess constructs such as burnout and clinician–patient relationships, they were not designed to evaluate the perceived impact of patient behavior on clinician performance and decision‐making in implant dentistry. Second, the study includes a multicountry sample of clinicians with diverse professional backgrounds, enhancing the applicability of the findings across different clinical settings. Third, the combined assessment of behavioral, emotional, and perceived performance variables allows for a more comprehensive evaluation of the clinician experience and supports the identification of indirect relationships between variables.

Several limitations should be acknwledged. The cross‐sectional design precludes causal inference. Furthermore, because the study evaluated clinicians’ perceptions of patient encounters, the findings reflect subjective experiences of clinician–patient interactions and should not be interpreted as objective measures of patient behavior alone. Moreover, because all index encounters were pre‐selected as challenging, the distribution of patient‐behavior ratings and the observed associations may not be representative of implant encounters more broadly. In addition, these perceptions may have been influenced by individual clinician‐related factors such as professional experience, communication skills, and practice environment. Participants were recruited through convenience sampling, and uneven country representation may limit the generalizability of the findings. Although the questionnaire was developed specifically for the objectives of this study and reviewed by an expert panel, formal psychometric validation was beyond the scope of this exploratory study. Finally, the inclusion of clinicians with varying roles and levels of implant experience may limit discipline‐specific interpretation.

Future research should further investigate the relationships between patient behavior, clinicians’ emotional responses, and perceived clinical performance using longitudinal and experimental study designs with more standardized assessment of clinician–patient encounters. Such studies could help clarify the temporal nature of these associations and evaluate their potential impact on clinical outcomes and practitioner well‐being. In addition, the development and validation of more comprehensive instruments, together with the evaluation of communication and behavioral management training, may further strengthen research in this field. The integration of objective outcome measures would also provide valuable complementary data to self‐reported measures. Finally, the observed associations highlight the importance of incorporating communication and management of challenging patient interactions into implant education, which may enhance clinician preparedness while supporting clinician well‐being and patient‐centered care.

## 5 Conclusion

Among implant encounters perceived by clinicians as challenging, patient behavior was associated with increased clinician emotional burden and perceived interaction difficulty, whereas case difficulty showed comparatively limited associations. Although patient behavior was not directly associated with lower perceived clinical performance, greater emotional burden was linked to increased perceived impact of challenging encounters and avoidance of similar patients. These findings highlight the potential relevance of interpersonal and human‐factor dimensions in implant practice and suggest that patient‐related factors may contribute to clinician experience and treatment‐related decision‐making.

## Author Contributions

**Aspasia Pachiou:** concept/design, data analysis/interpretation, drafting article, statistics, data collection, methodology, software, project administration. **Viviane Laura Humm:** critical revision of article, approval of article, data collection. **Nadja Naenni:** critical revision of article, approval of article. **Eleana Kontonasaki:** validation, critical revision of article, approval of article. **Ronald E. Jung:** critical revision of article, approval of article. **Daniel S. Thoma:** concept/design, critical revision of article, approval of article, methodology, validation, supervision.

## Funding

The authors have nothing to report.

## Ethics Statement

This study was conducted in accordance with the principles of the Declaration of Helsinki. Participation was voluntary, and the survey was completed anonymously. No personally identifiable information was collected. According to the Swiss Human Research Act (HRA), research involving fully anonymized questionnaire data without identifiable personal information does not require approval from a cantonal ethics committee. Therefore, formal ethical approval was not required.

## Consent

Patient consent was not applicable because this study did not involve patients or patient data.

## Conflicts of Interest

The authors declare no conflicts of interest.

## Permission to Reproduce Material From Other Sources

Not applicable.

## Supporting information


Supporting File

## Data Availability

The data that support the findings of this study are available on request from the corresponding author. The data are not publicly available due to privacy or ethical restrictions. The data that support the findings of this study are available from the corresponding author upon reasonable request.
