# Supplementary material for: Association Between Patient Behavior and Clinician Emotional Burden, Perceived Performance, and Decision‐Making in Implant Dentistry: A Cross‐Sectional Study Among Clinicians
Source: Clin Exp Dent Res. 2026 Sep 8;12(5):e70450. doi: 10.1002/cre2.70450 (PMC13552864; doi:10.1002/cre2.70450)
Supplement: Supplementary file 1 [file CRE2-12-e70450-s001.docx]

**Questionnaire**

**Section 1. Clinician Background Information**

**Q1. What is your clinical role?**
☐ General dentist
☐ Specialist in Prosthodontics/Implantology
☐ Specialist in Periodontology/Oral Surgery
☐ Resident (in any specialty)
☐ Other: ______________________

**Q2. Years practicing dentistry (including residency):**
☐ <3 years
☐ 3–5 years
☐ 6–10 years
☐ 11–20 years
☐ >20 years

**Q3. In what type of setting do you primarily work?**
☐ Private practice
☐ Academic/University clinic
☐ Hospital-based setting
☐ Other: ______________________

**Q4. Monthly implant cases (surgery or prosthetics):**
☐ 1–2
☐ 3–5
☐ 5–10
☐ >10

**Q5. What is your age?**
☐ Under 30
☐ 30–39
☐ 40–49
☐ 50–59
☐ >60

**Q6. What is your gender?**
☐ Female
☐ Male
☐ Prefer not to say

**Q7. In which country do you currently practice?**

**Section 2. Clinical Experience During This Implant Procedure**

*Please answer the following questions based on your most recent implant patient whose attitude or behavior you found challenging.*

**Q8. To help us group your responses anonymously, please enter the last 4 digits of your phone number. Use the same code each time you complete the survey.**

**Q9. How would you rate the difficulty of this procedure?**
☐ Very easy ☐ Easy ☐ Neutral ☐ Difficult ☐ Very difficult

**Q10. How would you rate the patient’s behavior?**
*(Consider communication, cooperation, and emotional demeanor.)*
☐ Very cooperative ☐ Cooperative ☐ Neutral ☐ Somewhat difficult ☐ Difficult

**Q11. How would you rate this patient’s behavior compared to your typical implant patients?**
☐ Much easier than usual ☐ Easier than usual ☐ About the same ☐ Slightly more difficult ☐ More difficult than usual

**Q12. How satisfied are you with your clinical performance today?**
☐ Very satisfied ☐ Satisfied ☐ Neutral ☐ Dissatisfied ☐ Very dissatisfied

**Section 3. Influence of Patient-Related Factors**

*To what extent did patient-related factors (behavior and attitude) influence the following?*

**Q13. Subjective dental outcome**
**Q14. Objective dental outcome**
**Q15. Clinical decision-making**
**Q16. Treatment time**
**Q17. Perceived efficiency**
**Q18. Approach to the next patient**
**Q19. Clinical decisions in case of non-compliance**
**Q20. Emotional exhaustion after the appointment**
**Q21. Clinical focus during the procedure**
**Q22. Tendency to avoid scheduling certain patients due to stress**

**Response scale (for Q13–Q22):**
☐ Not at all ☐ Slightly ☐ Moderately ☐ Significantly ☐ A great deal

**Section 4. Interaction With the Specific Patient**

*Please indicate your level of agreement with the following statements regarding your interaction with this patient.*

**Q23. I find it hard to communicate with this patient.**
**Q24. I feel tense during the interaction with this patient.**
**Q25. I feel this patient is manipulative or tries to control the situation.**
**Q26. I feel frustrated while treating this patient.**
**Q27. I feel that whatever I do, this patient is not satisfied.**
**Q28. I am glad when this appointment ends.**
**Q29. I find myself disliking this patient.**
**Q30. I feel this patient does not respect my professional judgment.**
**Q31. I feel that this patient challenges my authority or treatment decisions.**
**Q32. I find this patient difficult to work with.**

**Response scale (for Q23–Q32):**
☐ Strongly disagree ☐ Disagree ☐ Neutral ☐ Agree ☐ Strongly agree
